# Assessing a Native American collegiate volleyball program’s motivational climate

**DOI:** 10.3389/fpsyg.2026.1575654

**Published:** 2026-04-16

**Authors:** Joseph L. Claunch, Mary D. Fry, Theresa C. Brown

**Affiliations:** 1 Department of Educational Psychology, University of Kansas, Lawrence, KS, United States; 2 School of Nursing and Health Studies, University of Missouri Kansas City, Kansas, MO, United States

**Keywords:** caring climate, coaching education, goal orientation, qualitative, task-involving

## Abstract

**Introduction:**

This study explored how assessment can guide the development of a motivational climate program for a collegiate Native American volleyball team, using qualitative methods to inform a future collaborative effort grounded in achievement goal perspective theory (AGPT) and a caring framework.

**Methods:**

Guided by action research principles, the researchers collaborated with the head coach (*N* = 1) and athletes (*N* = 15) to co-construct the purpose and identify priorities for a future motivational climate collaboration.

**Results:**

Findings revealed that many of the athletes were overcoming challenging backgrounds while striving to play collegiate volleyball; volleyball provided an important stress outlet; success was predominately defined through interpersonal relationships; athletes’ preferences matched task-involving climate features; the team was in a state of transition; and finally, while highly skilled, the team craved team bonding.

**Discussion:**

Assessments with Native American athletes are particularly crucial because they provide insight into cultural values and experiences that may inform the development of supportive, culturally relevant programs that enhance athlete well-being and performance in sport. The findings from this assessment served as a foundation to build a comprehensive motivational climate collaboration framed by applicable research in AGPT and a caring climate framework.

## 1 Introduction

Creating supportive and inclusive environments within team sports is essential for fostering positive outcomes for athletes. Researchers have identified the motivational climate as an important theoretical framework shaping athletes’ experiences and overall team performance, with the benefits of a task-involving and caring climate fostering intrinsic motivation, team cohesion, and athlete well-being (Amaro et al., 2023; Chicau Borrego et al., 2021; Claunch and Fry, 2016; Fry and Moore, 2019; Gano-Overway and Fry, 2024; Gano-Overway and Sackett, 2019; Hodge et al., 2014; Hogue, 2024). The few intervention studies designed to influence perceptions of the motivational climate in sports and physical activity settings have proved successful, effectively equipping coaches with strategies that lead to significant positive outcomes for athletes (e.g., Duda et al., 2013; McLaren et al., 2015; Smith et al., 2007). Yet, these interventions seldom account for the unique backgrounds of the athletes they aim to support, a critical oversight, particularly for Native American athletes, whose cultural and historical contexts profoundly influence their sports participation related outcomes. Given the cultural emphasis on community and interconnectedness among many Native American traditions (Johnson et al., 2023; Jumper-Reeves et al., 2014), exploring these dynamics hold promise for informing assessments that may guide any motivational climate collaboration opportunities designed to enhance Native American athletes’ sport experiences. In addition, this approach aligns with the science-to-practice framework utilized in sport psychology, which emphasizes culturally respectful and contextually relevant strategies that reflect the lived experiences of the communities involved (Martens, 1979; Schinke et al., 2024).

### 1.1 Goal orientation and climate features

Within achievement goal perspective theory (AGPT) (Nicholls, 1989), goal orientations determine how athletes define success in their sport and provide valuable insights into the types of strategies they may use to achieve their goals. A highly task-oriented athlete defines success based on personal effort and self-improvement, while a highly ego-oriented athlete measures success through social comparisons or relative performance. A strong task orientation leads athletes to pursue success through factors they can control, such as giving maximum effort, while a strong ego orientation directs their focus to aspects they may not be able to influence, such as their abilities relative to their peers (Duda, 2013).

A significant body of research has connected a strong task-goal orientation with adaptive achievement strategies, including believing high effort and cooperation are key to success, as well as a greater tendency to persist when challenged (for reviews, see Fry and Moore, 2019; Harwood et al., 2015). A strong ego orientation, on the other hand, has been associated with maladaptive motivational patterns, such as increased sport dropout, particularly when athletes perceive their abilities to be lacking. Some evidence suggests that athletes high in both task and ego orientations may gain performance advantages, particularly at elite levels; however, these benefits occur only when a strong task orientation mitigates the maladaptive effects of ego involvement (e.g., anxiety), highlighting the broad value of task orientation (Lochbaum et al., 2016).

Based on Nicholls’ AGPT, Ames (1992) proposed that the social environment plays an important role in shaping individuals’ goal orientations, with the criteria used to define success determining whether athletes perceive a task- or ego-involving climate on their sport teams. In a task-involving climate, success is defined by giving high effort, making personal improvements, demonstrating cooperation, and viewing mistakes as part of learning. Conversely, in an ego-involving climate, success is defined as being a top-performing athlete, leading to preferential treatment for only the best, encouragement of intra-team rivalry, and punishing mistakes (Seifriz et al., 1992). Nicholls (1989) maintained that fostering task-involving characteristics while de-emphasizing ego-involving conditions was pivotal for enhancing individuals’ long-term motivation. A task-involving climate in sport promotes greater athlete motivation by emphasizing effort, improvement, and mastery leading to increased enjoyment and persistence and encouraging athletes to focus on personal growth over social comparisons (Fry and Moore, 2019; Harwood et al., 2015).

In tandem with the motivational climate, sport psychology researchers emphasizing the caring climate (Noddings, 1984, 2003) must also be considered to foster optimal outcomes, and they define the caring climate as spaces where athletes feel invited, safe, supported, and valued (Newton et al., 2007). Newton and colleagues contend that a caring and task-involving climate are complementary and can set athletes up to thrive in sport, and researchers have supported the distinct yet related aspects of both climates in sport and physical activity settings (Duda and Appleton, 2016; Fry et al., 2020; Hogue, 2024). When athletes perceive caring qualities exhibited by the team, they report benefits including increased effort, enjoyment, intrinsic motivation, and commitment to their sport (Fry and Gano-Overway, 2010; Fry et al., 2020; Gano-Overway and Fry, 2024; Iwasaki and Fry, 2013).

### 1.2 Motivational climate interventions

Establishing a positive and supportive environment grounded in theory-based approaches is essential for fostering adaptive motivational responses and enhancing athlete development in sports. AGPT (Nicholls, 1989) and the caring climate (Newton et al., 2007; Noddings, 1984, 2003) are particularly valuable frameworks to consider, as both emphasize a focus on individual growth rather than comparison to others and promote mutual support and collaboration to ensure positive outcomes for athletes. Several case studies with collegiate and elite sport teams provide evidence that coaches can foster caring, task-involving (CTI) principles, resulting in a culture of accountability, enhanced athlete development, and team cohesion (Gano-Overway and Sackett, 2019; Hodge et al., 2014).

Coaching education designed to enhance the motivational climate can translate into positive results for athletes. For instance, Smith et al. (2007) designed a 75-min workshop educating basketball coaches on ways to foster a task-involving climate in a youth league (i.e., the Mastery Approach to Coaching), with athletes of trained coaches reporting significantly less sport performance anxiety. A similar intervention design was employed with youth soccer coaches, with greater team cohesion reported by the athletes working with the trained coaches (McLaren et al., 2015). Similarly, caring climate interventions have yielded positive outcomes in sport settings. For example, Newton et al. (2007) found that youth in a summer sports program reported greater enjoyment, motivation, and engagement in activities when coaches were trained to promote caring climate principles.

While the results from motivational climate interventions have been encouraging, initial assessments have not been described in the current motivational climate intervention research, an important phase within action research from which interventions should evolve (Beckmann and Kellmann, 2003). Initial assessments are particularly important when working within a community, ensuring that interventions are developed with and alongside participants rather than delivered to them (Andrews et al., 2019). To address these gaps, adopting an action research approach allows for a more iterative and collaborative process, ensuring that interventions are directly informed by the specific contexts and needs of the athletes and communities they aim to support.

### 1.3 Action research framework

Action research involves a partnership between participants and researchers in a cyclical process of assessment, planning, implementation, and evaluation, making this framework particularly valuable for developing context-specific interventions that are both practical and responsive to the unique needs and experiences of the target population (Argyris et al., 1985). Moreover, action research is aligned with the science-to-practice framework where practitioners both apply and contribute to scientific knowledge while honoring the cultural and contextual uniqueness of the communities they serve (Martens, 1979; Schinke et al., 2024). Central to action research design is that participants must actively engage in the investigative process, rather than having a design imposed upon them (Herr and Anderson, 2005).

There are important ethical reasons for utilizing an action research framework with Native American participants as historically, Native Americans have had little representation in the research process. Consequently, Western concepts have been embedded in theoretical frameworks which have historically imposed White cultural norms onto Native American populations (Lomawaima and McCarty, 2002; Smith, 1999). Even more egregious, there are documented instances of the scientific community exploiting, misrepresenting, and damaging the reputations of Native American research participants (Cochran et al., 2008). For these reasons, many Native American communities hold a heightened distrust of researchers and the Western scientific community in particular (Smith, 1999), further highlighting the need for research approaches that honor Native American worldviews and support community control to build trust and accountability (Johnson et al., 2023). Historical and ongoing inequalities in resources and land dispossession continue to shape the experiences of Native American peoples in the United States (Lomawaima and McCarty, 2002), including those involved in athletics. Using an action research approach allows us to collaborate with these communities to better understand how these structural conditions influence life in and beyond sport, while co-creating responses that respect culture, context, and community sovereignty (Johnson et al., 2023).

One advantage to action research is the framework’s emphasis on prioritizing the voices and increasing the contribution of participants in the research process (Cochran et al., 2008). Action research fosters a more inclusive and collaborative approach, something especially important to acknowledge and respect Native Americans’ cultural perspectives and lived experiences. In accordance with action research principles, the word *collaboration* replaces the word *intervention* to highlight the active role of participants throughout the project.

While the field of applied sport psychology has long recognized the important function of assessment in delivering appropriate sport psychology services, current research is limited in explaining the role of assessment in applying motivational climate research to practice. Given the call for more qualitative studies highlighting female athlete and coach perspectives (Gano-Overway and Sackett, 2019; Hodge et al., 2014) and given the lack of Native American representation in the sport psychology literature, the purpose of this study was to examine how qualitative assessment could inform the development of a motivational climate program for a collegiate Native American women’s volleyball team. In line with action research principles and the ethical standards of the sport psychology profession, a preliminary assessment prioritizing participants’ point of view was utilized through interview techniques. The feedback derived from the assessment served as the basis for developing a motivational climate program for the team for their upcoming season.

## 2 Materials and methods

### 2.1 Qualitative design overview

This study used a phenomenological approach (Sparkes and Smith, 2014) to best describe and understand the shared, lived experiences of athletes on an all-Native American volleyball team. Guided by a constructivist epistemology and relativist ontology, the research team approached the study with the belief that knowledge is built by athletes and coaches through their own experiences and interactions, and that reality is shaped by each athlete’s experiences, culture, and social context (Smith and McGannon, 2017; Smith and Sparkes, 2020; Sparkes and Smith, 2014). This ontological stance acknowledges that phenomenological findings are context-specific, and interpretations should be grounded in the participants’ shared experiences. The researchers’ goal was to listen closely to the athletes’ stories and build interpretations that were meaningful for them and relevant in the broader sport and cultural context.

This approach aligns closely with the principles of Indigenous Research Sovereignty, which emphasize collective benefit and the authority of communities to control and govern their own data knowledge (Hudson et al., 2023). Native American athletes are a cultural population often overlooked or misrepresented in research and when included, their voices and experiences are frequently framed from dominant (e.g., white) cultural perspectives rather than their own shared reality (Johnson et al., 2023). The research team’s approach aimed to honor and center the athletes’ authentic experiences and perspectives.

While working from an interpretive paradigm, the research team also acknowledged insights from Ronkainen and Wiltshire’s (2021) realist perspective on validity. The research team engaged in ongoing reflexivity, critically reflecting on how their personal backgrounds, identities, and assumptions may have shaped the research process and interpretation of findings. Throughout data collection and analysis, the team prioritized capturing the complexity of participants lived experiences by honoring their rich, nuanced narratives. Specifically, the research team’s perspective was informed by their background as motivational climate researchers and they acknowledge that they entered the study with the belief that fostering a CTI climate holds real, beneficial effects for athletes and teams. Thus, when analyzing data, the researchers recognized their belief in the CTI climate being a potential framework to apply while also letting the athletes tell their own stories about their current team experiences and the type of climate they believed would be of most value in the upcoming season. Consistent with Smith and McGannon (2017), multiple interpretations were welcomed, and reflexivity remained central throughout the research process. The ultimate goal was to generate findings that would be meaningful and useful not only for the upcoming motivational climate collaboration (Claunch et al., 2026), but for the broader audience of coaches, athletes, and others engaged in sport.

Finally, to ensure rigor and trustworthiness in this qualitative study, the research team followed best practices for qualitative research in sport and exercise psychology, as outlined by Smith and McGannon (2017). This included maintaining transparency across all stages of the research process, with clear documentation of study design, data collection procedures, and analytic decisions. Ethical considerations (e.g., informed consent, confidentiality, shared decision making) were upheld throughout the research process to foster a collaborative relationship grounded in trust and cultural sensitivity (Johnson et al., 2023).

### 2.2 Collaboration invitation

During the spring volleyball season, the lead researcher was contacted by the head coach of a Tribal College and University (TCU) women’s volleyball team. The coach was concerned that her athletes were not maximizing their performance potential and believed this was in large part due to some of the higher performing players reacting negatively to errors made by less experienced players during competition. From the coach’s perspective, these older players’ frustrations adversely affected the younger players’ self-confidence; moreover, the coach perceived the younger players were reluctant to discuss their feelings with their teammates. The coach asked the lead researcher for strategies to manage athletes’ frustrations and negativity without impacting their playing intensity. The coach also wanted to help younger athletes play with more assertiveness and confidence, particularly those who presented as more introverted. In their discussions about a collaboration using an action research framework, the lead researchers proposed, and the head coach agreed, that initiating a comprehensive assessment of the athletes’ perceptions of the motivational climate would be a valuable starting point.

The lead researcher met with the head coach prior to the end of their spring season to define the collaboration logistics, review the purpose of the assessment as part of the collaboration design, discuss measurement options, and provide a packet of possible assessment instruments (questionnaires, interview questions). Additionally, the head coach’s input was sought to better understand what she wanted to learn about her team from the assessment. Institutional Review Board (IRB) approval was granted by both the lead researcher’s institution and the TCU site.

### 2.3 Assessment design

To ensure all athletes’ voices were represented, each athlete was invited to one-on-one interviews with the lead researcher (Herr and Anderson, 2005). To invite the team to interviews, the lead researcher met with the entire team 1 week prior to the conclusion of the spring season to share his background and explain the purpose of the assessment, how their information would be used, and the informed consent procedures (Marshall and Rossman, 2013). Athletes were assured their participation was strictly voluntary, they could refuse to participate and/or withdraw at any time, their responses would be kept confidential, and their participation would not affect their status on the team. The head coach also expressed her support for the assessment during this meeting.

As this study was framed within a phenomenological approach to explore athletes’ lived experiences, the athletes’ voices were prioritized throughout the assessment. While action research is oriented toward increasing the involvement of participants in the research process, participants’ level of investment can vary considerably based on situational factors. The athletes in this study had limited time to invest and therefore did not participate in the writing, transcribing, and data analysis; however, the lead researcher sought their input through interviews and shared summary results to determine priorities for future action. In addition, notes from the researcher’s interactions with the head coach were integrated with the interview transcripts to provide additional insight into the team’s overall impressions of the motivational climate and possibilities for collaboration. Although this reflects a more flexible application of action research principles, the study design still emphasized collaboration, cultural responsiveness, and the co-construction of knowledge in ways that were consistent with both the athletes’ needs and the broader qualitative framework.

### 2.4 Participants

The sample size for the collaboration was limited to the number of athletes on the team, reflecting a convenience sample aligned with the study’s goal of authentic engagement within this specific team context. To protect the anonymity of the participants, limited descriptive information is provided. Further, the participants chose pseudonyms used in this study.

#### 2.4.1 Head coach

The head coach self-identified as Native American, having a master’s degree, being a former NCAA Division-1 athlete, and using she/her pronouns. The coach had over 12 years of coaching experience, including 9 years of head coaching experience at multiple levels (i.e., NCAA Division-1, NAIA, high school). At the time of the assessment, she was in her sixth season with a TCU team.

#### 2.4.2 Athletes

The TCU athletes (*N* = 15; aged 18–23 years) self-identified as Native American, representing seven different federally recognized tribes, each tribe having distinctive cultural orientations. Nine of the athletes grew up on a reservation and the other six athletes grew up in towns bordering a reservation. The group included athletes in their first (*n* = 4), second (*n* = 6), third (*n* = 1), and fourth (n = 4) years with the team.

All athletes who were invited to participate consented to the study. To maximize the depth of the data, the lead researcher was embedded within the team throughout the season, attending practices, games, and team activities, and conducting multiple interviews with each athlete. This approach, consistent with best practices for thematic analysis and qualitative rigor (Braun and Clarke, 2019; Guest et al., 2006), allowed for continual engagement and reflection, helping to capture the range of experiences and perspectives within the team.

#### 2.4.3 Researcher/sport psychology practitioner

The lead researcher joined the volleyball team over the duration of the study in the dual roles of researcher and sport psychology practitioner (Merriam, 2009). The lead researcher self-identified as Native American, a former collegiate-athlete, former coach, and using he/him pronouns. At the time of the assessment, he was a sport psychology doctoral student. Although the researcher was an outsider to the study participants, he was very familiar with their athletic program, which helped him establish a relationship more quickly with the participants than if he were a complete unknown.

### 2.5 Data collection

As already noted, researchers bring pre-existing belief systems and theoretical perspectives that can shape the research process (Merriam, 2009). The researchers entered this study with two main interests: (1) exploring ways the motivational climate shapes collegiate Native American athletes’ sport responses, and (2) collaborating with the volleyball team to identify strategies to optimize their motivation. These interests, alongside a background in motivational climate research and a belief in the potential value of the CTI climate, likely influenced both interactions with participants and interpretations of the data (Smith and Sparkes, 2020). While it was impossible for researchers to enter studies completely value-free, the research team took deliberate steps to ensure rigor by promoting transparency, self-awareness and trustworthiness throughout the study (Smith and McGannon, 2017). These efforts included the lead researcher sharing personal disclosures to build trust and rapport, conducting interviews designed to honor participants’ lived experiences, maintaining detailed documentation through a research journal, and continuously considering data trustworthiness (Smith and McGannon, 2017; Smith and Sparkes, 2020).

#### 2.5.1 Personal disclosure

During the athletes’ introductions to the lead researcher, he discussed his tribal affiliation, background, and interests in working with the team as a researcher and a sport psychology Certified Mental Performance Consultant (CMPC) in training. This personal disclosure was important to building trust and rapport with the participants. The lead researcher also informally observed the team’s final spring practice and tournament match, allowing him to become more familiar with the team dynamics while also helping participants become more comfortable with his presence within the team environment.

#### 2.5.2 Interviews

Semi-structured interviews (approximately 25–30 min) with each athlete were conducted on site, audio recorded, and transcribed verbatim (Merriam, 2009). The semi-structured questions (Table 1) were designed to gain insight into the following factors: athletes’ backgrounds, definitions of success in volleyball motivational preferences, and perceptions of the team environment. The open-ended structure of the interviews was intentionally selected to encourage the interviews to be guided by the athletes’ perspectives, while also minimizing the risk of interviewer bias or the possibility of the researcher asking leading questions to elicit responses aligned with the guiding theories (Lewis-Beck et al., 2003; Merriam, 2009). Additionally, questions were chosen to elicit athletes’ definitions of success, allowing the research team to consider the relevance of AGPT and caring climates to future collaboration designs.

**Table 1:** Initial interview questions for assessment.

Semi-structured interview questions
1. What is important for me to know about your background to better understand you?
2. Why do you play volleyball?
3. How would you define success for yourself in volleyball?
4. How can someone like a coach best motivate you in volleyball?
5. What are your thoughts about how to best motivate your teammates?
6. How would you describe your relationship with your teammates?
7. How would you describe your relationship with your coach?
8. What are the strengths of your current volleyball team?
9. What is an area that you want your team to improve upon for the upcoming season?

#### 2.5.3 Research journal

The lead researcher’s interactions and reflections with the head coach were collected in a field note journal. The field notes were transcribed and integrated with data from the interviews to provide a more comprehensive account of the assessment phase of the motivational climate collaboration (Maxwell, 2005). The researcher and coach had frequent conversations that the researcher included in the field notes. These notes captured several types of interactions, including: (1) the researcher offering positive feedback to the coach on her efforts to reinforce a CTI climate; (2) shared reflections between the researcher and coach on athletes’ responses to the team environment; and (3) collaborative brainstorming on strategies to continue embedding CTI climate features into daily practices. The field notes served as a valuable log of the evolving collaboration, helping the researchers make sense of ongoing interactions and adding depth to the interview data. By capturing reflective conversations and collaborative planning, field notes were an important part of shaping and guiding the motivational climate collaboration (Smith and Sparkes, 2020; Sparkes and Smith, 2014).

#### 2.5.4 Data trustworthiness

To support trustworthiness and rigor within the constructivist and phenomenological framework guiding this study, multiple strategies were used to ensure the interpretation of the data accurately reflected participants’ lived experiences. First, open-ended questions were chosen to allow participants’ points of view. Second, the lead researcher offered frequent participant check-ins, providing participants with copies of interview transcripts for reactions, corrections, and additional insights. Third, the research team met frequently in a peer review process to discuss and agree upon themes (Marshall and Rossman, 2013). Fourth, the final themes were shared with participants to elicit feedback and to ensure themes accurately captured participants’ experiences and perspectives.

### 2.6 Data analysis

The six-phase method for identifying, analyzing, and reporting themes described by Braun and Clarke (2006, 2019) was used to guide the assessment data analysis. Consistent with a constructivist and phenomenological approach, the goal was to identify patterns of meaning that reflected participants’ lived experiences, while also considering relevant theoretical constructs. First, the research team became familiar with the data by reading through the transcribed notes and research journal multiple times. Second, the team inductively coded the transcripts for repetitive words, phrases, and statements, allowing emergent themes to surface. Inductive coding was conducted to allow meaningful themes to emerge from participants’ own language and perspectives. In a second cycle, deductive coding was performed using *a priori* codes informed by relevant AGPT and caring climate research (Merriam, 2009).

Third, inductive and deductive codes were integrated and categorized by primary themes and sub-themes. The decision to use both inductive and deductive analytic strategies was intentional, as inductive coding ensured themes were grounded in the athletes’ lived experiences and cultural meanings, while deductive coding allowed the research team to examine how emergent themes aligned with or extended established AGPT and caring climate constructs. Together, this approach allowed the research team to remain grounded in participants’ perspectives while also considering how those perspectives connected to established motivational climate frameworks.

Fourth, the team met to refine the themes, until reaching the fifth stage of agreeing upon a final list of the most relevant themes based on the research objectives. The sixth and final stage involved the research team incorporating the themes into a final written report which was shared with the participants to invite feedback and ensure the interpretation resonated with their experiences. Participant feedback during the member-checking process confirmed that the themes resonated with their experiences. Minor wording adjustments were made to better reflect participants’ intended meanings, and no substantive disagreements required removing themes. Throughout, the research team remained attentive to the co-constructed nature of knowledge while staying mindful of the unique cultural context, relationships involved and the setting where the athletes’ experiences took place (Smith and McGannon, 2017; Smith and Sparkes, 2020).

## 3 Results

Analysis of the participants’ interviews and the head coach research journal revealed six themes: (1) overcoming personal challenges, (2) volleyball as a stress coping outlet, (3) defining success in volleyball, (4) motivational climate preferences within the sport, (5) a team in transition, and (6) talented athletes who lack team bonding. The coach’s input provided contextual insight into team dynamics, supported triangulation of athletes’ perspectives, and informed the interpretation of themes without overriding the athletes’ voices.

### 3.1 Overcoming personal challenges

For historical (e.g., dispossession of ancestral lands) and situational (e.g., limited access to resources) reasons, Native American communities continue to be disproportionally affected by a range of negative health, social, and economic conditions (Centers for Disease Control and Prevention, 2024). These disparities were reflected in the narratives that the athletes provided. We felt including these narratives was essential for how the athletes’ broader life experiences intersected with their athletic engagement, and for ensuring that the collaboration was grounded in the realities of the athletes’ lives while respecting their authority to share how their stores are represented in this research (Hudson et al., 2023).

While their challenges varied, over half of the athletes (58%) disclosed family-related adversities impacting their lives. For instance, Jane shared:

My background has been kind of rough…I came from a single mother. From like my junior to senior year there was a lot of family deaths going on. My mom was going through a lot because my father passed away that year so she kind of went crazy. She didn’t know how to grieve I guess, so that took its toll on me. I felt like I was the mom for my siblings. She’s an alcoholic, um, I come from a family where everybody drinks except my grandma. It’s taken over a lot of their lives.

Nissa also emphasized an upbringing that was shaped by substance abuse and the loss of a parental figure:

I come from basically nothing. I raised myself, my dad died, my mom was alcohol and drug addicted and she didn’t take care of me so I bounced from family to family. So therefore, I grew up pretty fast and um, I guess that really shaped who I am.

The hardships the athletes experienced were not isolated events from the past, but ongoing stressors affecting their day-to-day living. For example, Maggie described her current circumstances:

My mom drinks a lot and she has been in and out of jail since my sophomore year in high school. That’s when she was first gone for like a really long time. And then she came back and it has kind of been weird since then. And my sister has been downhill from there. My mom and them are always fighting and she has to deal with it all by herself and I am all the way here…. I’m afraid of what’s going to happen to them. Like I want to be mad at my mom so many times, but I’m just afraid I will lose her and be mad. And then my sister, oh my god that girl. Like I don’t even know what to say to them, like I’m here.

It was significant that athletes were willing to share difficult aspects of their personal history in these interviews as many commented they do not discuss these issues with others. Nissa shared, “I kind of isolate myself away from everything because there are certain things I go through and am going through that I do not think other people understand.” Patricia explained she suffered from post-traumatic-stress but clarified, “I do not tell a lot of people because I just do not like having pity, I do not want it; I do not want to be a victim anymore. I do not want to be looked at like that either.” Similarly, Jane commented,

That was hard just telling you because I haven’t talked about that kind of personal stuff and my coach doesn’t know any of that, but I don’t seek sympathy and I don’t seek attention for that kind of stuff. I don’t want people to feel sorry for me or anything.

Although Jane did not share parts of her personal history with the coach, the coach was aware many of her athletes were dealing with ongoing hardships. The coach explained,

I usually ask questions about, like, what is going on and then there is a breakdown, crying, and the story comes out…It is exhausting and I do want to be there to help them, but some things I don’t know how to help. And some of them need counselors beyond my abilities that I can really offer.

Despite the seriousness and difficulty of life circumstances that were described, most of the athletes were functioning at a high level in school and on the volleyball court. Multiple athletes had 4.0 grade point averages, and all were academically on track to graduate. Additionally, some did not describe any hardships presently or in the past. Penelope provided advice for professionals planning to work with Native American athletes, “We all come from different areas and even our experience, especially at our levels, and even in certain situations, how we adapt to what is going on around us. We are all different; I guess our mindsets are different.” This quote underscores the diversity of experiences among Native American athletes who are each shaped by their individual histories and tribal affiliations, while further highlighting that Native Americans do not share a single, uniform experience (Leavitt et al., 2015).

### 3.2 Volleyball as a stress coping outlet

Participants were asked to identify their reasons for playing volleyball and while several motives emerged, the most prominent was volleyball provided an outlet to cope with life stressors. For instance, Nissa responded,

An outlet. Like I said I was brought up with nothing and that made me angry as a child, like very angry and I was a bully for a while and then I met this teacher who like changed my whole perspective on it. She asked me about my story… so I told her and she was like, “You know what, I know you’re mad, but that’s not the way to be. You need to find an outlet; you need to do something.” I got into drawing and all this and then my teacher talked to my grandparents and she was like, “Well, she needs an outlet cause this anger is building up and she’s turning into something that she’s not, because she’s a smart girl.” So, they put me in boxing and I liked that, but my grandma didn’t like the whole violent thing, like how it caused me to start fighting, like sticking up to people, you know. So they’re like, “Well, an alternative is a volleyball club for Natives.” So, I got into the volleyball club and then, I don’t know, my whole life changed and it felt like I had a place, because when you play volleyball you have a sense of belonging, so I felt like I belonged, and I had love like from my team and my coach.

While Nissa’s stress outlet was to feel like she had a place where she belonged, other athletes indicated a need for a stress outlet that provided distraction. For example, Jane commented, “Volleyball has helped a lot, um, emotionally; it’s a big stress reliever.”’ Another athlete, Maggie, explained, ‘If there is something going on at home then it was always volleyball that could just take everything off my mind. It’s really nice to know that I can focus on it and not worry about anything else. Similarly, Tasha described,

When it’s not my day I’m always excited to go to practice where I can just like cool off and blow everything off. Going to practice is just a place I can be where I don't have to think of anything.

Several other athletes said their stress outlet was to find a place to have fun. Penelope stated, “I like it! I mean it’s weird because you know how some girls say they love it? I do not know if I love it, but I do enjoy it. It is a way to clear my head and its fun.” Similarly, Raven commented, “If I’m having a stressful day, me and Maggie will be just like, ‘ok, let us go play’ and just have fun doing that.” Overall, exploring the athletes’ motives provided the researchers with a foundation to structure the collaboration design.

### 3.3 Defining success in volleyball

As sport holds different meanings across cultures, it was important to explore how athletes defined success as a way to assess the relevancy of utilizing AGPT and the caring climate to frame the collaboration project. A few athletes gave definitions consistent with an ego-orientation, as they emphasized demonstrating, rather than developing, ability. For instance, Penelope explained:

I want to be known for volleyball too, like a great player. I plan to try to get the all-conference player and scholarship athlete, and I want to try and get my name on the banner. That is one of the biggest things I want to try for.

A few other athletes leaned more toward a task-orientation, focused on developing ability. For example, Jane described success in volleyball as, “To work harder, to be the best I can be. I do not see myself being like a cocky player or anything, I just want to grow and keep growing.”

However, most athletes did not give an immediate definition of success that clearly reflected task- or ego-orientations, and instead, reflected a primary desire to enhance interpersonal relationships on the team. Lesha explained, “I would define success by respecting my teammates and becoming more comfortable with my coach. I hope to become better when I get done with my 2 years being here and, you know, bringing it back to my community.” Note while Lesha’s comments reflect the task-orientation of wanting to become better at volleyball, her desire to become better is tied to her aspiration of bringing the sport to her community. In another example, Raven acknowledged that winning is important to feeling successful, yet her definition puts primary emphasis on the team relationships:

I guess winning is a big part, everyone wants to win, but I really look at the other stuff, like with the team, I always try to make sure the team is close. Coming from my recent high school team we weren’t always the winning team…but it got me focused on more of the other things, like making my improvement and also the team, how I could help them get together and improve our chemistry and stuff because I really think that's a big part.

Likewise, Maggie defined success in volleyball as, “Helping as much as I can. It does not really bother me if someone is better than me. It’s ok with me as long as I know I am helping in some way, like contributing to the team.” The athletes prioritized positive interpersonal relationships and caring for teammates, reflecting collectivistic cultural values common in many Native American communities (e.g., extended family networks, community responsibility; relationship quality; collective well-being; Hudson et al., 2023; Jumper-Reeves et al., 2014), which likely influenced their definitions of success in volleyball as aligned with shared cultural values over individual achievements.

### 3.4 Motivational climate preferences within the sport

When asked about best ways to motivate themselves and teammates, the athletes’ responses revealed preference for a task-involving, caring approach. Multiple athletes said they wanted feedback on how to improve, consistent with a defining feature of a task-involving climate (Nicholls, 1989). Angela shared that she wanted discussion around, “Goals. Like this is where you want to be so this is what you have to do to get there. Just the thought of it and bringing it up is motivating to me.” Raven preferred a coach who would, “Tell me or tell a person how much potential they have and if they keep working, they could like grow into something more. I’m thinking that is one of my best motivations from a coach.”

Also in line with a CTI climate, multiple athletes expressed a preference for supportive, constructive feedback when discussing mistakes and a desire to want to improve. For example, Suzanne explained:

If there’s a problem, come up to me and be like, “Hey Suzanne, you’re not doing what you need to do right now.” And more of a talking tone, not a scolding tone. And not in front of everybody either because sometimes that gets embarrassing too. So, if it’s more on a one-on-one basis.

Conversely, Suzanne described a punitive method for responding to mistakes consistent with an ego-involving climate, which she found detrimental to her play and sense of well-being:

I don’t like yelling. I don’t take that well. I can handle it if a coach is a yeller or I’m in trouble, but sometimes it doesn’t help me. It makes me feel like I’m screwing up and then I feel like “Ah I messed up, like okay, I get it, I’m messing up. I’m sorry.” And then I get frazzled. And then after that it gets hard for me to get my self-esteem back up. That’s what it is. It kind of hurts my self-esteem.

In line with AGPT (Nicholls, 1989; Seifriz et al., 1992), Suzanne’s comments highlight how mistake-contingent punishment, a defining feature of an ego-involving climate, had deleterious effects on her self-esteem. Suzanne desired a more task-involving, caring climate, where mistakes are expected, viewed as part of the learning process, and discussed in a supportive and constructive manner.

Similarly, Tasha explained that to remain motivated after an undesirable performance she needed, “Encouragement to keep trying, that’s about it or after every mistake we come in and talk in a little circle for just a few seconds and encourage each other to play better the next time.” When Tasha was prompted to describe how encouragement would help her respond after making a mistake, she indicated, “My energy level would get a boost if they [the coach and fellow athletes] did a little more encouragement on the court; that would make me want to push myself more, but getting negative feedback, it just puts me down a little.” It was clear from the athletes’ responses they were more motivated by constructive responses to mistakes, an approach aligned with AGPT’s emphasis on viewing mistakes as part of the learning process (Seifriz et al., 1992; Nicholls, 1989) and caring climates’ emphasis on support and collaboration (Newton et al., 2007; Noddings, 1984, 2003).

### 3.5 A team in transition

In discussing the status of current team dynamics, many athletes contrasted the past fall team with the current spring team, stating the current spring team’s atmosphere was improved. Raven shared,

Everyone is in good standing with each other, not like in the past. The past was bad, it was kind of frustrating; like it was mostly like the older ones that…looked down on us and that made it kind of frustrating throughout the whole season.

Jane provided additional details on the changes that the team had recently undergone:

I’m closer to them (teammates) this year than I have been this previous season. A lot of older players were kind of rude and they weren’t respectful enough, so a lot of the teammates that I came in with didn’t bond with them. They didn’t try to get to know them, but who would want to with that kind of attitude, you know?

Some athletes noted that the previous fall team had struggled with divisions between the older and younger players. Jane went on to explain that the division created, “Cliques; you had the older players, and you had the girls who kept to themselves.”

This environment had deleterious effects for multiple players. For instance, Nissa commented, “Previous players really intimidated me, like telling me on the sideline, ‘You’re not going to get playing time, you are a freshman!’” When Nissa was prompted to describe how these older players comments affected her, she stated, “I kind of just stepped back and took in what they said. You’re a freshman, you are not going to play. So my level of work just, you know, sidelined basically and I just accepted it.” The former players’ devaluation of Nissa and other players’ inexperience reflects an ego-involving climate that, according to AGPT, likely undermined their motivation by failing to make them feel valued and an integral team member. These comments also underscore the absence of a caring climate, leading Nissa to feel she lacked both value and influence to affect any change to her situation.

Similarly, Maggie explained how her treatment made her question if she would continue playing:

My first year here I didn’t want to play anymore for a while. They were mean and then other people were like, well it’s just your first year of college sports. My friend’s dad was like, “I played college football and they used to mess with me the first year, but it gets better and the years go on and everybody is cool with each other after that.” But it was so hard. I didn’t want to do anything.

The advice Maggie received from the former collegiate athlete further normalized the negative behavior causing her to contemplate quitting. When prompted to describe the fall team dynamics, Maggie answered:

Not supportive, I guess. They [seniors] expected us to know stuff they have learned over four years, you know? And they wouldn’t really show us; they were just like, “You’re supposed to do this!” It made me not want to go to practice or lift or anything. I red-shirted my first year and after that I didn’t know if I wanted to play anymore.

The athletes’ comments in this study underscore the critical need for a caring climate, emphasizing the significant influence peers have on shaping other athletes’ perceptions of the climate, as well as the negative effects when caring is perceived to be lacking.

### 3.6 Talented athletes who lack team bonding

In the final questions of the interview, the athletes were asked to assess the team’s strengths and areas for future improvement. A number of team strengths were shared such as commitment to a common goal, experience, and offense. The most prominent strength was the team’s high skill level. As Rachel explained, “I guess our skill level is better and I think we know how to play, but we just need to incorporate it all together.”

While discussing areas for improvement, athletes expressed a desire for a stronger off-court connection, reflecting the lack of a caring climate highlighted in other comments. For instance, Caitlin explained,

We’ll be together and we’ll create our bonds, but then again, we’ll separate and we’ll go back to our little cliques and we’ll stay distant for a while, especially out of season. I mean we’ll still see each other, but we could do more together in the off-season.

Rachel also indicated that the team could be closer, especially in the off-season:

I’d say by doing team bonding, because everybody just seems to like, do their own thing after, like off the court. I mean that’s cool and I don’t mind, but I think we could use that extra time to come together.

Likewise, Nissa explained:

We all should have a connection and I feel like we don’t have that as a team. A lot of people would rather go out with one or two people on the team, rather than, do something together and like socializing… As a volleyball player, I want to have the team bond.

Like many of the athletes on this team, Suzanne perceived a lack of cohesion with her teammates, explaining, “When it comes to the team, I do not really associate with a lot of them. I’m close with Jane. She’s the only one I’d reach out to for anything.” Suzanne went on to share her preference, “I like to be able to know that I can go to them for anything. Yeah, just to know that they kind of have my back.” This sentiment reflects the lack of caring highlighted in other interviews, where the absence of mutual support and trust undermines the sense of community with the teams.

Given the strong desire for off-court team bonding (i.e., which the research team defined as wanting camaraderie and social connection among team members through activities and interactions beyond formal sport practice), the lead researcher prompted athletes for recommendations to enhance relationships across the team. Many of the athletes’ ideas focused on increased opportunities to develop social bonds. Patricia recommended, “I think we should just do like a lot more team bonding stuff, like we could all like go do something like laser tag.” Similarly, Lesha recommended, “One thing that I would really like to see though, is us having a group session maybe one weekend we would hang out and do something other than volleyball… like a get together.” Raven advocated for hands on team bonding activities versus sitting in a class:

I like having team bonding through like activities. There’s this one place we went to in high school; it’s called Broken Arrow, it’s like a bible camp. They were really trying to show us team bonding and they did that through these games that in the end showed the lesson that you had to work together to get this done. Yeah, this group is really wanting to learn through hands on activities, [rather] than like sitting in a class or something.

In team sport, success is often determined by a team’s ability to combine the skills of individuals and direct them toward a common goal. These athletes believed they were skilled enough to be successful; however, there was consensus that stronger social bonds would be to the benefit of everyone.

## 4 Discussion

The purpose of this study was to qualitatively assess the motivational climate for a Native American collegiate volleyball program in preparation for a collaboration opportunity. As action research emphasizes the importance of inclusivity and valuing participants’ voices, qualitative assessment with Native American communities are especially important to address historical exploitation and mistrust in the research process. By completing an assessment before offering any sport psychology services with the team, the researchers aimed to respect cultural perspectives and promote ethical collaboration design while trying to avoid imposing Western norms. Qualitative assessment methods, including interviews and feedback opportunities, allow researchers to emphasize the voices of athletes and coaches to ensure the resulting program reflects their values, needs, and lived experiences (Andrews et al., 2019). This approach is particularly important when working with Native American athletes, who have historically been underrepresented in sport psychology research and whose cultural perspectives may differ from mainstream approaches (Claunch and Fry, 2016; Hudson et al., 2023; Johnson et al., 2023; Jumper-Reeves et al., 2014; Leavitt et al., 2015). This assessment approach yielded valuable and important context-specific information to incorporate into any future work with the team. One on one interviews with the athletes and ongoing informal conversations with the coach illuminated how multiple factors consistent with CTI and ego-involving features impacted their motivation, as well as factors beyond theoretical tenets of AGPT and caring climates.

### 4.1 Preparing for athletes’ needs beyond performance outcomes

Many of the athletes indicated they were navigating ongoing family and personal hardships. These accounts highlight challenges that deserve thoughtful attention. While all athletes face struggles and difficulties, the severity and scope of the trauma experienced by athletes on this team reflect complexities that may exceed what the typical collegiate coach is prepared to support (Hertzler-McCain et al., 2023). It is remarkable these athletes were able to balance their commitments to volleyball and school, given the magnitude of the challenges they described. This underscores the importance of conducting a comprehensive assessment as part of action research, as this exercise helped the research team better understand and appreciate how to design the collaboration.

Acknowledging the presence of family and community-related adversities in these athletes’ lives was not intended to define them by hardship, as is often the case in Native American research (Hudson et al., 2023; Leavitt et al., 2015). Consistent with our action research approach and the principles of Indigenous research sovereignty (Hudson et al., 2023), we aimed to represent the athletes’ experiences in ways that reflected their perspectives and the contexts surrounding the challenges they faced on and off the court. Including these stories was not meant to reduce their identities to stereotypes or victimhood, a common criticism of Native American research (Leavitt et al., 2015), but to show the realities that shaped their participation in sport and to honor the lived experiences that informed this collaboration.

The coach can play a pivotal role in creating a climate that helps build athletes’ resiliency when faced with adversity (Gano-Overway and Sackett, 2019; Hodge et al., 2014; Vitali et al., 2015). Yet, the head coach in this study admitted to feeling inadequate at times to address all her athletes’ needs. While it is not uncommon for coaches to serve in a variety of capacities, the coach’s comments illuminate the complex role coaches play and their important role in creating psychological safety on a team (Vella et al., 2024). Like the coach, many of the challenges that the athletes expressed were beyond the boundaries of the lead researcher’s professional competence as well. Performing an assessment before collaboration is critical to identifying athlete concerns. Knowing ahead of time the athletes’ needs, the lead researcher was able to better prepare by developing a referral network of local professionals available to those presenting major challenges beyond the scope of their athletic relationship (Association for Applied Sport Psychology, 2012).

In addition to family and personal hardship, the majority of athletes used their volleyball playing as an outlet including for experiencing fun and a sense of belonging, and reduced stress. In particular, the stress relief provided is consistent with previous research among collegiate athletes showing how athletic participation can buffer stress by providing a distraction (Kimball and Freysinger, 2003; Kleiber et al., 2002). Research has consistently shown a positive relationship between perceptions of a CTI climate and reduced levels of stress, anxiety and increased resilience, well-being, while perceptions of an ego-involving climate have been linked to increased levels of stress and anxiety (Fry and Moore, 2019; Hogue, 2024; Scott et al., 2021; Smith et al., 2007; Vitali et al., 2015). Perhaps the connection between stress and task-involving climate is not surprising, as this climate’s emphasis on one’s own effort, improvement and teamwork (rather than social comparison and winning at all costs), allows athletes to feel more competent in their support, autonomous in their actions and more supported overall. These factors, in turn, help make the sport experience less stressful (Nicholls, 1989; Ruiz et al., 2019). To honor the needs of this particular team, the researchers learned that designing a motivational climate program focused on enhancing the stress-relief benefits of volleyball while minimizing its stress-inducing aspects was a key priority for the collaboration design.

A final consideration about how to prepare for the athletes’ needs came from Penelope who reminded the researchers that every athlete on the team should be treated as an individual with unique and diverse interests and needs. Despite the existence of hundreds of Native American tribes in the United States, each with distinct cultural traditions, Native Americans have historically been misrepresented as one homogenous group (Leavitt et al., 2015). The team was made up of inter-tribal Native American women and therefore, the collaboration design must allow for potential similarities and differences between each individual athlete. Previous research highlighting coach strategies highlight the importance of coaches recognizing every athlete has individual needs (Gano-Overway and Sackett, 2019). While many of the athletes shared similarities such as tumultuous upbringings, the researchers were reminded to be cautious of insensitive stereotyping or drawing inferences about Native Americans in general in future collaboration work.

### 4.2 Adding interpersonal relationships to definitions of success

To determine whether an AGPT and caring climate framework fit with any upcoming collaboration designs, the researchers asked athletes to provide their definitions of success in volleyball (i.e., goal orientations). A few athletes expressed a primary preference for feedback on how to continue to improve and to address mistakes using supportive and encouraging instruction. Their desire to keep improving is a key feature of both a task orientation and a task-involving climate, which are associated with self-driven motivation. In these environments, athletes experience success through personal progress, leading to personal growth and greater enjoyment of the sport (Amaro et al., 2023).

Likewise, other athletes expressed their dislike of more ego-involving climate characteristics, where punishment of mistakes was detrimental to their sense of well-being. The coach and teammates attitudes, beliefs, and treatment of one another can shape the motivational climate and influence athletes’ perceptions of success (Smith et al., 2009). Just as past research in sport has linked perceptions of an ego-involving climate with lower self-esteem (Harwood et al., 2015; Reinboth and Duda, 2004), some athletes in the current study recognized how ego-involving characteristics brought heightened awareness of inadequacy, ultimately impacting their self-worth.

While task- and ego-involving climate characteristics emerged, the majority of responses reflected a belief that success meant positive interpersonal relationships with their teammates, a finding consistent with a caring climate. Sports and relationships with teammates can greatly impact athletes’ social well-being, feelings of belonginess, and perceptions of social support (Hall et al., 2017; Inoue et al., 2015), and athletes’ perceptions of a caring climate have been consistently linked to better social and emotional development and greater team cohesion (Gano-Overway and Fry, 2024; Gano-Overway and Sackett, 2019). Some of the athletes even highlighted how their caring sport environment provided an important social context for prosocial involvement, a connection that has been made in previous research with underserved youth (Gould et al., 2012; Newton et al., 2007).

A possible explanation for these athletes’ emphasis on quality relationships to define success may relate to the collectivistic cultural values prevalent in many tribal communities, where community, relationships and collective well-being are often prioritized; though it is important to recognize the considerable diversity of experiences and orientations among Native American individuals and tribes (Hudson et al., 2023; Schinke and Hanrahan, 2009). In their work exploring goal orientations among African American athletes, Gano-Overway and Duda (1999) concluded that athletes “come to the playing field with a tendency toward a particular motivational perspective that is in line with their cultural values” (p. 559). Thus, it is possible that the collectivistic orientation of many Native American athletes’ communities helps shape definitions of success in volleyball. Given the priority these athletes placed on developing quality relationships with their teammates and evidence that fostering a caring climate is related to enhanced peer relationships, the collaboration design should include strategies for fostering caring relationships.

### 4.3 Designing a CTI collaboration

While motivational climate interventions often target coach behaviors (Claunch and Fry, 2016; McLaren et al., 2015; Newton et al., 2007; Smith et al., 2007), many of the athletes’ in the current study emphasized the influential role that their peers play in shaping the quality of their volleyball experiences. Research on the athlete’s role in shaping the motivational climate is limited, yet studies have revealed that like coaches, athletes do wield a powerful influence over their peers’ perceptions of the climate (Ntoumanis and Vazou, 2005; Vazou et al., 2005). Thus, any plan to optimize the motivational climate for this team must account for the athletes as well as the coach’s role, providing the athletes with opportunities to engage in activities that could help them foster more caring relationships with their teammates and contribute to the task-involving climate.

In addition to peer relationships, it was clear from the athletes’ interviews they preferred positive and constructive responses to help them learn from mistakes. Their preferences were in line with the theoretical position outlined in AGPT, which posits that more adaptive outcomes will result when mistakes are viewed as a normal part of the learning process (Nicholls, 1989). The athletes’ comments also correspond to a substantial body of literature linking mistake-contingent encouragement and instruction with a range of beneficial outcomes, including high levels of perceived competence, self-esteem, enjoyment, and lower levels of anxiety (Black and Weiss, 1992; Conroy and Coatsworth, 2006; Fry and Moore, 2019; Smith et al., 2007).

A final theme that emerged through the assessment was a desire to heal from hurt caused by previous team dynamics. Several athletes felt their team contributions were devalued by previous players and their actions were consistent with an ego-involving climate where athletes are treated differently based on athletic abilities. Facilitating an open conversation within the team about past experiences of feeling devalued might help further support the development of a more CTI climate. From an AGPT perspective, fostering adaptive motivation in sport is in part contingent upon making all team members feel like they play an important role, and as evident by Nissa’s comments, motivation to perform can suffer when this does not occur. Previous researchers have found a range of maladaptive responses in ego-involving climates, including lower sport commitment and stronger intentions of dropping out (Fabra et al., 2021; Hall et al., 2017). The athletes’ clearly desired a collaboration design meant to strengthen social bonds both on and off the court, a finding consistent with past research linking social and task cohesion with better sport performances (Carron et al., 2002).

### 4.4 Limitations and future directions

This study sought to integrate cultural relevance with research on task-involving and caring climates to design an effective team sport collaboration experience. While this study involved an assessment, an often-overlooked step in action research, considered the perspective of an underrepresented population in the sport psychology literature, and employed an interview format allowing for valuable exploration of complex human behaviors and experiences, the qualitative design is not without limitations. A key aspect of qualitative research is the co-constructed nature of knowledge, recognizing that findings emerge through the interactions that occur between the participants’ experiences and the researchers’ interpretations (Smith and Sparkes, 2020; Merriam, 2009). Rather than viewing subjectivity as a limitation, this study embraced it by practicing transparency and ongoing reflection throughout the research process (Smith and McGannon, 2017). The research team followed a rigorous, six-phase method for thematic analysis (Braun and Clarke, 2006, 2019) and actively involved participants by sharing summaries and inviting feedback to ensure interpretations resonated with their perspectives (Marshall and Rossman, 2013). These steps were taken to honor the complexity and uniqueness of athletes’ experiences while balancing trustworthiness and credibility.

The small sample sizes typical of qualitative studies limit their generalizability to the broader population. In addition, replicability is difficult as this assessment was context-specific to this team and this particular time. However, while the findings of this study may be specific to the collaboration interests of this team, there is value in following a similar approach, especially with Native American populations where efforts to establish trust are particularly salient. Ethical concerns also arise when dealing with sensitive topics, requiring careful measures to protect participants’ privacy and emotional well-being. As evident through the interviews, athletes shared several personal hardships impacting their overall wellbeing, which must be accounted for in any future collaborative work with the group.

The American Psychological Association (2006) recognizes evidence-based practices in psychology are most effective when they are responsive to contextual factors, such as the participants’ strengths, challenges, culture, and preferences. While previous motivational climate interventions have been employed with clinical expertise, the extant research has not explored the important role that context, culture, and preference play in integrating the research findings in applied sport settings. Consistent with the evidence-based practices, the current study highlighted the value of a qualitative assessment which honor participants’ voices and allow for collaborative approaches that are culturally appropriate and contextually relevant.

### 4.5 Practical implications

This study underscores the importance of conducting thorough qualitative assessments to inform collaboration designs, particularly when working with underrepresented populations such as Native American collegiate athletes. By prioritizing the voices of the athletes and respecting their cultural perspectives, the researchers were able to identify key motivational and relational factors that influence their experiences, from recognizing personal challenges that impact their play, to using volleyball as a stress reliever, to recognizing how the athletes define success, and the importance of team interpersonal relationships to the athletes’ motivation.

Building on these insights, coaches and sport psychologists can translate this understanding into practice by creating a CTI climate that supports positive peer interactions and attends to athletes’ individual and cultural needs. Programs can reinforce this approach by incorporating athlete perspectives in program design, ensuring that relational experiences are valued alongside performance outcomes. Athletes too can benefit from these findings by reflecting on how they contribute to a supportive team climate, putting forth their best effort, and communicating openly with coaches and teammates about their needs. These strategies help interventions be both contextually relevant and effective in supporting athletes’ motivation, well-being, and team cohesion.

The findings emphasize the need for collaboration designs that foster trust, enhance social bonds, and promote a task-involving and caring climate, while remaining sensitive to the individual and collective needs of the athletes. While qualitative research naturally embraces subjectivity, this approach allowed for nuanced, culturally sensitive insights that can guide ethical and effective sport psychology practices in similar settings.

## Data Availability

The datasets presented in this article are not readily available because approval from the Institutional Review Board prevents us from sharing participants’ data. Requests to access the datasets should be directed to mfry@ku.edu.
